# Performance of dye-affinity beads for aluminium removal in magnetically stabilized fluidized bed

**DOI:** 10.1186/1477-044X-2-5

**Published:** 2004-08-26

**Authors:** Handan Yavuz, Ridvan Say, Müge Andaç, Necmi Bayraktar, Adil Denizli

**Affiliations:** 1 Department of Chemistry, Biochemistry Division, Hacettepe University, Ankara, Turkey; 2 Department of Chemistry, Anadolu University, Ankara, Turkey; 3 Faculty of Medicine, Urology Department, Hacettepe University, Ankara, Turkey

## Abstract

**Background:**

Aluminum has recently been recognized as a causative agent in dialysis encephalopathy, osteodystrophy, and microcytic anemia occurring in patients with chronic renal failure who undergo long-term hemodialysis. Only a small amount of Al(III) in dialysis solutions may give rise to these disorders.

**Methods:**

Magnetic poly(2-hydroxyethyl methacrylate) (mPHEMA) beads in the size range of 80–120 μm were produced by free radical co-polymerization of HEMA and ethylene dimethacrylate (EDMA) in the presence of magnetite particles (Fe_3_O_4_). Then, metal complexing ligand alizarin yellow was covalently attached onto mPHEMA beads. Alizarin yellow loading was 208 μmol/g. These beads were used for the removal of Al(III) ions from tap and dialysis water in a magnetically stabilized fluidized bed.

**Results:**

Al(III) adsorption capacity of the beads decreased with an increase in the flow-rate. The maximum Al(III) adsorption was observed at pH 5.0. Comparison of batch and magnetically stabilized fluidized bed (MSFB) maximum capacities determined using Langmuir isotherms showed that dynamic capacity (17.5 mg/g) was somewhat higher than the batch capacity (11.8 mg/g). The dissociation constants for Al(III) were determined using the Langmuir isotherm equation to be 27.3 mM (MSFB) and 6.7 mM (batch system), indicating medium affinity, which was typical for pseudospecific affinity ligands. Al(III) ions could be repeatedly adsorbed and desorbed with these beads without noticeable loss in their Al(III) adsorption capacity.

**Conclusions:**

Adsorption of Al(III) demonstrate the affinity of magnetic dye-affinity beads. The MSFB experiments allowed us to conclude that this inexpensive sorbent system may be an important alternative to the existing adsorbents in the removal of aluminium.

## Background

About 8% of the Earth's crust is comprised of aluminium. This element is the most abundant metal naturally present in air, soil and water. Consequently, environmental exposure to aluminium is potentially possible. Its ingestion is unavoidable since aluminium compounds are added not only to most water supplies but also to many processed foods and medicines. Aluminium is a known neurotoxicant. It enters the brain, where it contributes to some neuro-degenerative diseases including dialysis encephalopathy, osteomalacia, osteodystrophy, in particular those related to dialysis treatment of uremic subjects [1]. Only a small amount of Al(III) ions in dialysis solutions may cause these disorders. Aluminium may contribute to Alzheimer's disease [2]. Aluminium is also able to give rise to toxicity in the bones and hematopoietic system in humans [3].

Positively charged aqua and hydroxy-monomeric forms have been found to be the most toxic species of aluminium to living organisms in the terrestrial and aquatic environments [4]. Generally, aluminium sulphate is used as a coagulant in the treatment of water to help the removal of suspended matter and highly coloured humic substances [5], thus reducing the dose of chlorine later required to ensure satisfactory microbiological quality. Hence, potable water often contains high aluminium levels of natural origin and/or from the water purification process [6].

The selective removal of aluminium ions have been extensively investigated by applying several techniques [7-9]. Among them, the use of specific polymeric adsorbents has been considered as one of the most promising techniques [10,11]. Specific adsorbents consist of a ligand (e.g., reactive textile dye, ion-exchange functional groups or chelating agents) which interacts with the metal ions specifically, and a carrier solid matrix.

There have been several separation approaches performed under magnetic field [12]. The most well known technique is the magnetically stabilized fluidized bed. Magnetically stabilized fluidized bed exhibits combination of the best characteristics of both packed and fluidized bed. These include the efficient fluid-solid mass transfer properties, elimination of particle mixing, low pressure drop, high feed-stream solid tolerances, good fluid-solid contact, elimination of clogging and continuous countercurrent operation [13]. Especially, when dealing with highly viscous mediums contact with the magnetic adsorbent in a magnetically stabilized fluidized bed is desirable because of high convective transport rates. Recently, there has been increased interest in the use of magnetic adsorbents in biomolecule coupling and nucleic acid purification [14]. Magnetic adsorbents can be produced using inorganic materials or polymers. High mechanical resistance, insolubility and excellent shelf life make inorganic materials ideal as adsorbent. The main disadvantage of inorganic supports is their limited functional groups for ligand coupling. Magnetic adsorbents can be porous or non-porous [15]. They are more commonly manufactured from polymers since they have a variety of surface functional groups which can be tailored to use in different applications [16-22].

In the present study, we attempted to use alizarin yellow-attached magnetic poly(2-hydroxyethyl methacrylate) (mPHEMA) beads as specific adsorbent for aluminium removal from aqueous solutions in a magnetically stabilized fluidized bed. Al(III) adsorption on the alizarin yellow-affinity beads from aqueous solutions containing different amounts of Al(III) ions and at different pHs is reported here. Finally, reuse of the dye-affinity beads is also discussed.

## Materials and methods

### Materials

2-hydroxyethyl methacrylate (HEMA), was purchased from Sigma (St. Louis, MO, USA), and was purified by vacuum distillation under a nitrogen atmosphere. The comonomer, ethylene dimethacrylate (EDMA, Merck, Darmstadt, Germany) was used as the crosslinking agent. Magnetite particles (Fe_3_O_4_, diameter < 1 μm) were obtained from Aldrich (USA). Alizarin yellow (3,4-dihydroxy-9,10-dioxo-2-anthracenesulfonic acid, sodium salt mono-hydrate) was purchased from BDH (Poole, UK). All other chemicals were obtained from Merck as analytical grade. All water used in the adsorption experiments was purified using a Barnstead (Dubuque, IA) ROpure LP^® ^reverse osmosis unit with a high flow cellulose acetate membrane (Barnstead D2731) followed by a Barnstead D3804 NANOpure^® ^organic/colloid removal and ion exchange packed bed system.

### Preparation of magnetic PHEMA beads

Details of the preparation and characterization of the mPHEMA beads were reported elsewhere [23]. The mPHEMA beads were prepared by suspension polymerization. A typical suspension copolymerization procedure of mPHEMA beads was performed as below: The dispersion medium was prepared by dissolving 200 mg of poly(vinyl alcohol) (PVA; molecular weight: 50.000) within 50 ml of distilled water. The desired amount of 2,2'-azobisisobutyronitrile (AIBN) (0.06 g) was dissolved within the monomer phase 12.0/4.0/8.0 ml (EDMA/HEMA/toluene) with 1.0 g magnetite particles. This solution was then transferred into the dispersion medium placed in a magnetically stirred (at a constant stirring rate of 600 rpm) glass polymerization reactor (100 ml) which was in a thermostatic water bath. The reactor was flushed by bubbling nitrogen and then was sealed. The reactor temperature was kept at 65°C for 4 h. The temperature was then raised to 90°C and kept constant by a thermostated water bath during the polymerization time (2 h). After polymerization, the mPHEMA beads were separated from the polymerization medium. The residuals (e.g., unconverted monomer, initiator and other ingredients) were removed by a cleaning procedure. Briefly, beads were transferred into a reservoir, and washing solutions (i.e., a dilute HCI solution, and a water-ethanol mixture) were recirculated through the system which includes also an activated carbon column, to be assured that the magnetic beads were clean. Purity of the magnetic beads was followed by observing the change of optical densities of the samples (λ: 280 nm) taken from the liquid phase in the recirculation system, and also from the DSC thermograms of the magnetic beads obtained by using a differential scanning microcalorimeter (Mettler, Switzerland). Optical density of uncleaned magnetic beads was 2.63, but after the cleaning operation this value was reduced to zero. In addition, when the thermogram of uncleaned beads was recorded, it had a peak around 60°C. This peak might originate from AIBN, but after application of the cleaning procedure, no peak between 30–100°C was observed on the thermogram.

The dry density of the magnetic beads was measured with pycnometer by dispersing the dry beads in ethanol.

### Alizarin yellow attachment

Preparation and characterization of the alizarin yellow-attached mPHEMA beads were reported in our previous paper in detail [24]. In order to prepare the alizarin yellow-attached magnetic beads following procedure was applied. 5.0 g of dry magnetic beads was weighed and transferred into the SOCl_2 _(Carlo Erba, Italy) (10 ml). This reaction medium was boiled in rotary evaporator for 6 h. Then, 2.5 g alizarin yellow was dissolved in absolute ethanol (30 ml). Alizarin yellow-attachment process was performed in ethanol solution for 24 h. At the end of this reaction period, the alizarin yellow-attached beads were removed by filtration and washed with ethanol, water and tetrahydrofuran several times until all the unbound dye molecules were removed. The dye attached beads were stored at 4°C with 0.02% sodium azide to prevent microbial growth.

The leakage of the alizarin yellow from the dye-attached beads was investigated within the media at the selected pH in the range of 2.0–7.0. These media were the same which were used in the Al(III) adsorption experiments. The medium with the dye attached magnetic beads was stirred for 24 h at room temperature. Then, magnetic beads were separated from the medium, and the alizarin yellow concentration was measured in the liquid phase by spectrophotometry at 500 nm.

### Magnetically stabilized fluidized bed procedure

Al(III) adsorption studies were carried out in a magnetically stabilized fluidized bed. Beads suspended in pure water were degassed under reduced pressure (by using water suction pump) and magnetically stabilized into a column (10 cm × 0.9 cm inside diameter) equipped with a water jacket for temperature control. The vertically oriented magnetic field was produced by passing DC current through two modified Helmholtz coils (1.5 cm diameter × 2.5 cm thick) spaced 4 cm apart. At a current of 1.6 A (50 W), each coil produced a magnetic field of 40 Gauss. Equilibration of the column was performed by passing four column volumes of phosphate buffer (pH: 7.4) before injection of the Al(III) solution. In a typical adsorption system, 50 ml of the aqueous Al(III) solution was passed through the column containing magnetic beads, by a peristaltic pump for 2 h. After loading, the column was washed with deionized water to wash out Al(III) impurities. The concentrations of the Al(III) ions in the aqueous phases after the desired treatment periods were measured by using a graphite furnace atomic absorption spectrophotometer (AAS 5EA, Carl Zeiss Technology, Zeiss Analytical Systems, Germany). Deuterium background correction was used. Pyrolitic graphite coated tubes were used for AAS measurements. The instrument response was periodically checked with known Al(III) solution standards. The experiments were performed in replicates of three and the samples were analyzed in replicates of three as well. For each set of data present, standard statistical methods were used to determine the mean values and standard deviations. Confidence intervals of 95% were calculated for each set of samples in order to determine the margin of error.

In the first group of experiments, the flow rate of the aqueous solution (i.e., 50 ml of the solution with a Al(III) content of 50 mg/L) was changed between 0.5–3.0 mL/min. In the second group of experiments, Al(III) adsorption from aqueous solution was studied at different pH's (2.0–7.0). Adsorption isotherm was also obtained in the magnetically stabilized fluidized bed. Aqueous solutions containing different amount of Al(III) were used in these experiments. The changes in the Al(III) concentration with time was followed to obtain the adsorption curves. The amount of adsorbed Al(III) per dry magnetic beads was calculated by using the concentrations of the Al(III) in the initial solution and in the equilibrium.

### Desorption and repeated use

In all cases adsorbed Al(III) ions were desorbed using 0.1 M HNO_3 _solution. In a typical desorption experiment, 50 ml of the desorption agent was recirculated through the magnetically stabilized fluidized bed containing dye-affinity magnetic beads for 1 h. The concentrations of the Al(III) ions in the desorption medium were measured by using a graphite furnace atomic absorption spectrophotometer. The desorption ratio was calculated from the amount of Al(III) adsorbed on the magnetic beads and the final Al(III) concentration in the desorption medium. In order to test the reusability of the dye-affinity magnetic beads, Al(III) adsorption-desorption procedure was repeated ten times by using the same magnetically stabilized fluidized bed.

### Batch procedure

Adsorption of Al(III) from aqueous solution was also investigated in batch experiments. Aqueous Al(III) solution (50 ml) was treated with the magnetic dye-affinity beads at room temperature, in the flasks agitated magnetically at an agitation speed of 600 rpm for 2 h. The suspension was brought to pH 5.0 by adding sodium hydroxide and nitric acid. The pH was maintained in a range of ± 0.1 units until equilibrium was attained. Polymer amount was kept constant at 100 mg per 50 ml. Al(III) determination was performed in water sample in an atomic absorption spectrophotometer coupled to a graphite furnace atomiser. Adsorption values (mg/g) were calculated as the difference in Al(III) ion concentration of the pre- and post adsorption solutions divided by the weight of dry magnetic affinity beads.

## Results and discussion

### Characteristics of mPHEMA beads

mPHEMA beads (in the size range of 80–120 μm) carrying alizarin yellow were prepared as a specific affinity adsorbent for removal of Al(III) from the water which was used for preparation of dialysis solution. mPHEMA beads used in this study were prepared and characterized in our earlier study [24]. The main criteria of selection of PHEMA is due to its mechanical strength and chemical stability. With the goal of testing the mechanical stability of the magnetic beads, a sample of these magnetic beads was treated in a ball mill for 60 min. Negligible percentage of the sample was broken. The dry density of the magnetic beads was measured as 1.27 g/cm^3^. The magnetic beads are crosslinked hydrogels. They do not dissolve in aqueous media, but do swell, depending on the degree of cross-linking and on the hydrophilicity of the matrix. The equilibrium swelling ratio (the ratio of the volumes of the microbeads before and after swelling) of the beads used in this study is 34%. The simple incorporation of water weaken the secondary bonds within the hydrogels. This enlarges the distance between the polymer chains and causes the uptake of water. It should be mentioned that the water uptake properties of the mPHEMA beads did not change after Alizarin Yellow attachment.

After the attachment of the dye (i.e., alizarin yellow) the size of the swollen beads did not change, but the colour became dark yellow, which is a clear indication of the incorporation of the dye molecules in the structure of the mPHEMA microbeads. As shown in our previous paper, the dye molecules were attached to the mPHEMA beads by covalent bonding via hydroxyl groups [24]. The mPHEMA beads containing 208 μmol alizarin yellow/g polymer, which was the maximum amount of dye-attachment that we have reached, were used in this study. Alizarin Yellow release from the mPHEMA beads was also monitored continuously. There were no dye release in any of the adsorption and desorption media, which assured that the cleaning procedure used for removal of physically adsorbed alizarin yellow molecules from the mPHEMA beads was satisfactory.

### Column performance

**Figure 1:**
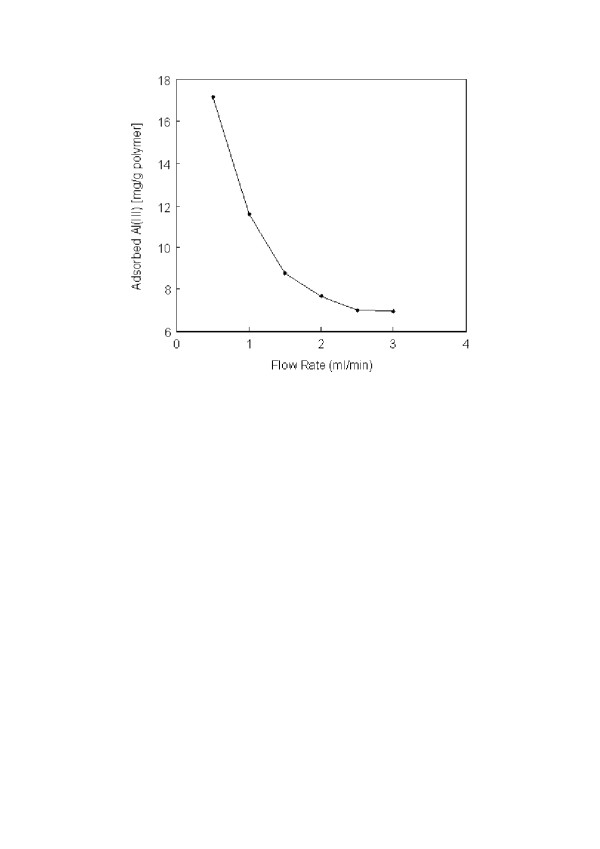
Effect of flow-rate on Al(III) adsorption. Alizarin yellow loading: 208 μmol/g; Al(III) concentration: 50 mg/L; pH: 5.0; T: 25°C.

The adsorption capacity at different flow-rates are given in Figure 1. The adsorption capacity decreased significantly from 17.2 mg/g to 6.9 mg/g polymer with the increase of the flow-rate from 0.5 ml/min to 3.0 ml/min. One of the explanation for such phenomenon would be a faster ligand-metal ion (i.e., alizarin yellow) dissociation rate compared to the association rate. Hence, the adsorbate (i.e., Al(III) ions) would pass through the magnetically stabilized column without adsorption at high flow-rate. Second explanation could be that the increased nonideal flow hydrodynamics of liquid phase and the solid phase for magnetically stabilized fluidized bed. These phenomena can be summarized by the increase of the axial dispersion coefficient in the axial dispersion model [25].

### Adsorption capacity

**Figure 2:**
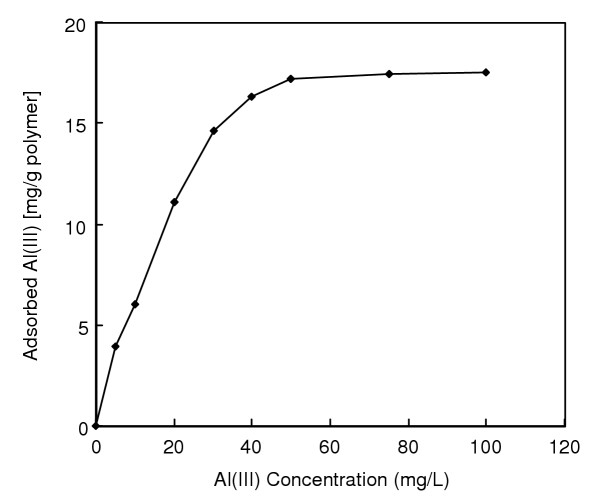
Effects of Al(III) concentration on Al(III) adsorption. Alizarin yellow loading: 208 μmol/g; Flow-rate: 0.5 ml/min; pH: 5.0; Adsorption time: 60 min; T: 25°C.

Figure 2 shows the adsorption profile of Al(III) ions. The amount of Al(III) ions adsorbed per unit mass of the polymer (i.e. adsorption capacity) increased first with the initial concentration of Al(III) ions then reached a plateau value at about an initial Al(III) ions concentrations of 50 mg/L, which represents saturation of the active attachment sites (which are available for Al(III) ions) on the beads. The maximum adsorption capacity of Al(III) ions was of 647 μmol/g (17.5 mg/g). Unit mass of the mPHEMA beads carries 208 μmol alizarin yellow which was found by elemental analysis. From the mass-stoichiometry, it seems that one attached alizarin yellow molecule interacts with around three Al(III) ions. Since alizarin yellow has seven coordinating sites of a single sulphur and six oxygen atoms, it can form a ternary complex which is coordinated with water molecules at vacant coordination sites of metal-alizarin yellow complexes.

It should be noted that the nonspecific adsorption (adsorption on plain mPHEMA beads) of Al(III) ions was relatively low (0.63 mg/g). mPHEMA beads do not contain ion exchange or chelating groups. Preferred coordination structure and preferred coordinating ligand atom may be utilized for this adsorption. Al(III) ions may interact with Oxygen atoms as the ligand. Diffusion of Al(III) ions into the swollen polymeric structure and retention in the pores may also contribute to this nonspecific Al(III) adsorption.

### Adsorption isotherms

An adsorption isotherm is used to characterize the interactions of each molecule with the adsorbent. In this case it provides a relationship between the concentration of the Al(III) ions in the solution and the amount of Al(III) ions adsorbed on the solid phase when the two phases are at equilibrium. The Langmuir adsorption model assumes that the species are adsorbed at a fixed number of well-defined sites, each of which is capable of holding only one molecule. These sites are also assumed to be energetically equivalent, and distant from each other so that there are no interactions between molecules adsorbed on adjacent sites.

Adsorption isotherms were used to evaluate adsorption properties. The Langmuir adsorption isotherm is expressed by Equation 1. The corresponding transformations of the equilibrium data for Al(III) gave rise to a linear plot, indicating that the Langmuir model could be applied in these systems and described by the equation:

Q = Q_max_. b . C_eq _/ (1 + bC_eq_)     (1)

where Q is the adsorbed amount of Al(III) (mg/g), C_eq _is the equilibrium Al(III) concentration (mg/mL), b is the Langmuir constant (mL/mg) and, Q_max _is the maximum adsorption capacity (mg/g). This equation can be linearized so that

C_eq_/Q = 1/(Q_max_. b) + C_eq_/Q_max_.     (2)

The plot of C_eq _versus C_eq_/Q was employed to generate the intercept of 1/Q_max_.b and the slope of 1/Q_max_.

The maximum adsorption capacity (Q_max_) data for the adsorption of Al(III) was obtained from the experimental data. The correlation coefficient (R^2^) was 0.989. The Langmuir adsorption model can be applied in this affinity adsorbent system. Maximum adsorption capacities determined using Langmuir isotherms show that dynamic capacity (25.3 mg/g) was somewhat higher than the batch capacity (12.6 mg/g). The dissociation constants for Al(III) were determined using the Langmuir isotherm equation to be 27.3 mM (MSFB) and 6.7 mM (batch system), indicating medium affinity, which was typical for pseudospecific affinity ligands.

### Effect of pH

**Figure 3:**
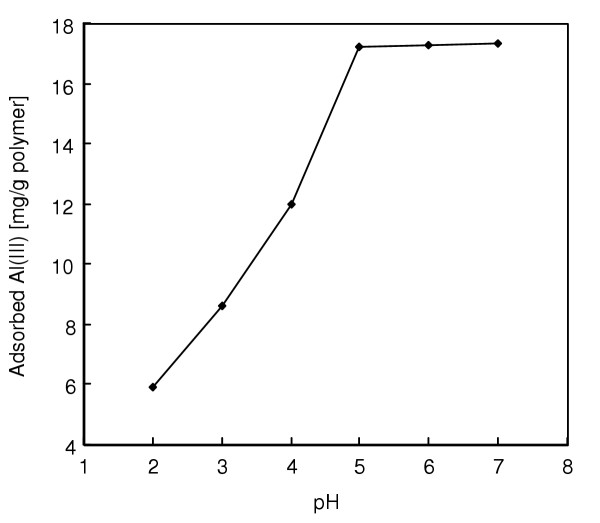
Effects of pH on Al(III) adsorption. Alizarin yellow loading: 208 μmol/g; Flow-rate: 0.5 ml/min; Al(III) concentration: 50 mg/L; Adsorption time: 60 min; T: 25°C.

Metal ion adsorption onto specific adsorbents is pH dependent. In the absence of complexing agents, the hydrolysis and precipitation of the metal ions are affected by the concentration and form of soluble metal species. The solubility of metal ions is governed by hydroxide or carbonate concentration. Hydrolysis of metal ions becomes significant at approximately pH 7.5–8.5. Therefore, in the present study, we changed the pH range between 2.0–7.0. The effect of pH on the Al(III) adsorption of this alizarin yellow-attached mPHEMA beads is also shown in Figure 3. The magnetic mPHEMA beads exhibited a low affinity in acidic condition (pH < 4.0), a somewhat higher affinity between pH 4.0 and 7.0. High adsorption capacities at around neutral pH values imply that Al(III) ions interact with dye molecules not only through the oxygen atoms by chelating, but also electrostatically through sulfonate groups, which are ionized at neutral pH.

### Competitive adsorption

Competitive adsorption of the metal ions from tap water in Ankara and dialysis water (reverse osmosis) was also investigated. The water containing different amounts of each metal ion was treated with dye beads in MSFB. Table 1 and 2 show the adsorbed amounts for each metal ion. The adsorption capacity of the dye-attached mPHEMA beads for Cu(II) and Al(III) ions was higher than that for other ions. But it should also be noted that the extent of adsorption of each type of metal ion is strongly dependent upon their relative concentrations within the medium.

**Table 1:** Aluminium removal from the tap water.

Metal Ion %	Concentration of Metal Ions (ng/ml)	Metal Ion Adsorption (μg/g)	Adsorbed Metal Ions (%)
Al(III)	80.1	40.3 ± 0.1	98.9
Fe(III)	32.1	10.8 ± 0.2	54.3
Cu(II)	145.3	28.3 ± 0.2	35.1
Cd(II)	0.05	nd	-
Pb(II)	0.03	nd	-
Zn(II)	20.4	1.5 ± 0.1	28.6

Adsorption Conditions: Flow-rate: 0.5 ml/min; pH: 5.0; T: 25°C, nd: not determined. Each experiment was repeated three times.

**Table 2:** Aluminium removal from dialysis water.

Metal Ion %	Concentration of Metal Ions (ng/ml)	Metal Ion Adsorption (μg/g)	Adsorbed Metal Ions (%)
Al(III)	18.96	9.46 ± 0.1	99.7
Fe(III)	0.05	-	-
Cu(II)	0.82	0.16 ± 0.01	40.0
Zn(II)	1.26	0.44 ± 0.01	69.8

Adsorption Conditions: Flow-rate: 0.5 ml/min; pH: 5.0; T: 25°C. Each experiment was repeated three times.

The World Health Organization (WHO) and the European Community (EC) guide values for Al(III) ions for tap water is 200 ng/ml [26,27]. Al(III) concentrations both in tap water and dialysis water are below this value. It should be noted that polymer treatment (i.e, adsorption) significantly decreases the metal content and these purified waters can be used safely especially for the preparation of dialysis solutions. Magnetic dye-affinity beads exhibits the following metal ion affinity sequence: Al(III) > Cu(II) > Fe(III) > Zn(II).

### Desorption and repeated use

**Figure 4:**
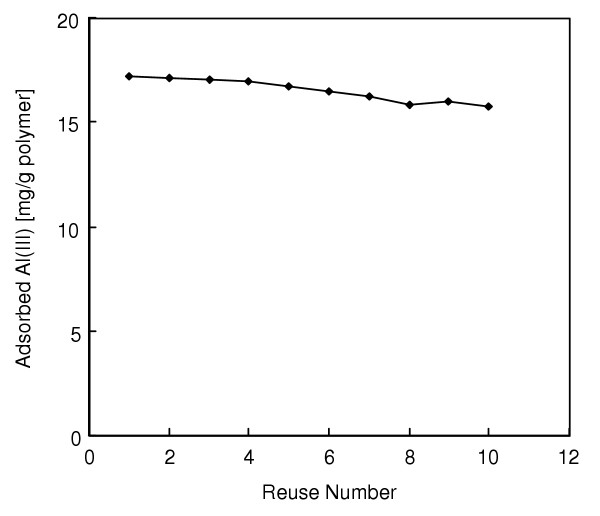
Repeated use of dye-attached mPHEMA beads. Alizarin yellow loading: 208 μmol/g; Flow-rate: 0.5 ml/min; Al(III) concentration: 50 mg/L; Adsorption time: 60 min; pH: 5.0; T: 25°C.

Desorption ratios were very high (up to 97.6%) with the eluant system and under conditions used. When HNO_3 _is used as a desorption agent, the coordination spheres of chelated Al(III) ions are disrupted and subsequently Al(III) ions are released from the solid surface into the desorption medium. Therefore, we conclude that HNO_3 _is a suitable desorption agent for the dye adsorbents, and allows their repeated use. In order to show the reusability of the dye-attached mPHEMA beads, adsorption-desorption cycle was repeated ten times by using the same sample of affinity adsorbent. As can be seen from Figure 4, adsorption capacities did not noticeable change during the repeated adsorption-desorption cycles.

### Comparison of magnetically stabilized fluidized bed and batch system

**Figure 5:**
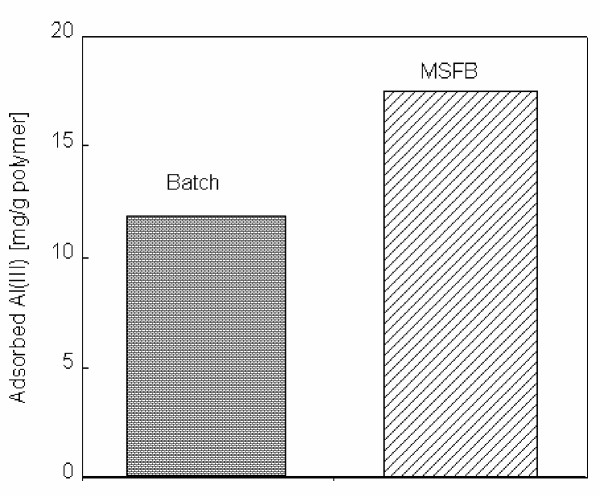
Comparison of MSFB and batch system. Alizarin yellow loading: 208 μmol/g; Flow-rate: 0.5 ml/min; Al(III) concentration: 50 mg/L; Adsorption time: 60 min; pH: 5.0; T: 25°C.

As can be seen in Figure 5, maximum Al(III) adsorption from aqueous solution is 11.8 mg/g for batch system and 17.5 mg/g for MSFB system. These results indicated that the adsorption capacity obtained in MSFB system is considerably higher than obtained in batch sytstem. This means, in equilibrium binding experiments, maximum capacity was 38.8% lower as compared to the value obtained in MSFB. This result could be explained in two ways. (i) The dye ligand-Al(III) dissociation rate in the batch system is higher than the association rate in the MSFB system. (ii) Alizarin yellow ligand is found both on the surface and in the pores of the magnetic beads. In the presence of flow, the Al(III) solution is forced from the surface into the pores thus eliminating the surface diffusion.

## Conclusions

The medical relevance of aluminium has stimulated the development of cost and time effective separation techniques including polymeric carriers. Magnetic adsorbents have several potential advantages over conventional adsorbents [28-32]. The magnetically stabilized columns require faster processing times and high flow-rates with a much lower operating pressure than a packed bed column. In this study, mPHEMA beads, in the size fraction of 80–120 μm, were produced by a dispersion polymerization of EGDMA and HEMA in the presence of magnetite particles. These novel magnetic beads were then successfully attached with reactive dye-ligand, namely alizarin yellow. The highest dye loading was 208 μmol/g. Al(III) adsorption capacity of the beads decreased with an increase in the flow-rate. The maximum Al(III) adsorption was observed at pH 4.0. Al(III) adsorption onto the mPHEMA beads was negligible (0.63 mg/g). Higher adsorption values (up to 17.5 mg/g) were observed using alizarin yellow attached mPHEMA beads for the adsorption of Al(III) ions from aqueous solutions. Al(III) ions could be repeatedly adsorbed and desorbed without significant losses in their adsorption capacities.
